# Medicinal Plant Extracts Targeting UV-Induced Skin Damage: Molecular Mechanisms and Therapeutic Potential

**DOI:** 10.3390/ijms26052278

**Published:** 2025-03-04

**Authors:** Chunhui Zhao, Shiying Wu, Hao Wang

**Affiliations:** 1 Key Laboratory of Plant Biotechnology of Liaoning Province, School of Life Sciences, Liaoning Normal University, Dalian 116081, China; zch@lnnu.edu.cn (C.Z.); wsymaodou@163.com (S.W.); 2 Lamprey Research Center, School of Life Sciences, Liaoning Normal University, Dalian 116081, China

**Keywords:** ultraviolet radiation, skin damage, medicinal plants, active ingredients, exosome-based therapy, drug delivery systems

## Abstract

The depletion of the ozone layer has intensified ultraviolet (UV) radiation exposure, leading to oxidative stress, DNA damage, inflammation, photoaging, and skin cancer. Medicinal plants, widely used in Traditional Herbal Medicine (THM), particularly in Traditional Chinese Medicine (TCM), have demonstrated significant therapeutic potential due to their well-characterized active compounds and established photoprotective effects. This review systematically evaluates 18 medicinal plants selected based on their traditional use in skin-related conditions and emerging evidence supporting their efficacy against UV-induced skin damage. Their bioactive components exert antioxidant, anti-inflammatory, DNA repair, and depigmentation effects by modulating key signaling pathways, including Nrf2/ARE-, MAPK/AP-1-, PI3K/Akt-, and MITF/TYR-related melanogenesis pathways. Moreover, novel drug delivery systems, such as exosomes, hydrogels, and nanoemulsions, have significantly enhanced the stability, bioavailability, and skin penetration of these compounds. However, challenges remain in standardizing plant-derived formulations, elucidating complex synergistic mechanisms, and translating preclinical findings into clinical applications. Future interdisciplinary research and technological advancements will be essential to harness the full therapeutic potential of medicinal plants for UV-induced skin damage prevention and treatment.

## 1. Introduction

Worldwide, humans are inevitably exposed to sunlight throughout their daily lives. While moderate sun exposure facilitates the synthesis of vitamin D, prolonged overexposure can result in significant skin damage. As the body’s primary sensory organ, the skin interacts directly with the external environment, and chronic sunlight exposure is associated with sunburn, photoaging, and even skin cancer. Consequently, repairing ultraviolet (UV)-induced skin damage has emerged as a critical topic in dermatological research.

Humans have recognized the potential threats of ultraviolet (UV) radiation to the skin since ancient times. Around 5000 BC, ancient Egyptians applied a mixture of rice bran, jasmine, and lupine to their skin to protect against UV damage. Later studies revealed that lupine extract could enhance skin protein synthesis and fortify the skin barrier, while jasmine extract exhibited antibacterial and anti-inflammatory effects and reduced melanin deposition [1,2,3,4]. The combination of these extracts effectively aids in repairing UV-induced skin damage.

With the rapid advancements in molecular biology, the late 1980s witnessed the introduction of novel technologies such as gene therapy, cell therapy, and exogenous enzyme therapy into the field of skin damage repair. These approaches have demonstrated substantial potential for application in skin repair. By the end of the 20th century, medicinal plant extracts had also begun to integrate with modern technologies, gaining extensive use in skin repair research. In recent years, emerging therapies, including nanotherapy, stem cell exosome therapy, 3D bioprinting, and cold plasma therapy, have also found increasing applications in repairing skin damage [5,6,7,8,9,10,11,12].

Medicinal plant extracts are bioactive substances derived from the collection, processing, and clinical application of plants. Currently, approximately 5000 medicinal plants have been identified, with active ingredients primarily comprising flavonoids, alkaloids, and other organic compounds, extensively applied in skin repair. In comparison to antibiotics and epidermal growth factors, medicinal plant extracts offer gentler therapeutic effects, facilitating skin repair via mechanisms such as promoting fibroblast proliferation and regulating inflammatory responses [13]. This review provides a comprehensive overview of medicinal plants with significant roles in treating UV-induced skin damage. These plants were selected for their widespread use in Traditional Herbal Medicine (THM), particularly in Traditional Chinese Medicine (TCM), for treating dermatological conditions. They have demonstrated efficacy in wound healing, with increasing research attention on their role in UV-induced skin damage. Their active compounds are well characterized, and their photoprotective effects have been substantiated in both in vitro and in vivo models.

## 2. Mechanisms of UV-Induced Skin Damage and Therapeutic Targets

### 2.1. Types of Ultraviolet Radiation and Their Impact on Skin

Ultraviolet (UV) radiation, a component of the electromagnetic spectrum with wavelengths ranging from 10 nm to 400 nm, is primarily emitted by sunlight. UV radiation is categorized into three types based on wavelength: A (UVA), B (UVB), and C (UVC). The ozone layer absorbs most UV radiation with wavelengths shorter than 300 nm, thereby protecting Earth’s organisms from harmful radiation [14]. Although UV radiation exhibits germicidal properties, it also poses significant risks to biological macromolecules such as DNA, RNA, and proteins. Chronic exposure to UV radiation causes cumulative damage to skin cells, heightening the likelihood of carcinogenesis or apoptosis [15].

#### 2.1.1. UVA

UVA, with a wavelength range of 320–400 nm, is classified as long-wave ultraviolet radiation. It has strong penetration capabilities and can pass through glass to reach the skin. UVA radiation intensity is affected by factors such as time of day, geographic location, and ozone layer thickness. Its intensity is lower in the morning and evening than at midday. About 70–80% of UVA radiation is absorbed by the epidermis, while the remainder penetrates the dermis, impairing the production of elastic fibers and collagen, which contributes to skin photoaging [16]. Short-term exposure to UVA can cause melanin deposition, skin aging, and pigmentation. Prolonged exposure induces the production of reactive oxygen species (ROS), which damage DNA and proteins, elevating the risk of skin cancer [17].

#### 2.1.2. UVB

UVB, with a wavelength range of 280–320 nm, has weaker penetration compared to UVA. It cannot penetrate glass and is easily absorbed by the ozone layer and atmospheric oxygen. Brief exposure to UVB aids in vitamin D synthesis, while prolonged exposure promotes the accumulation of ROS in the skin and impairs antioxidant enzyme activity, resulting in epidermal damage [18]. UVB induces DNA damage through the interaction of high-energy photons with DNA. While cells can repair such damage after brief exposure, prolonged exposure significantly increases the risk of skin cancer [19].

#### 2.1.3. UVC

UVC radiation, with a wavelength range of 200–280 nm, possesses high energy but low penetration power. It is largely absorbed by the Earth’s ozone layer. UVC is frequently employed for air sterilization and disinfection [20]. While prolonged exposure can induce skin erythema, desquamation, and photoaging, leading to skin cancer in severe cases, UVC radiation with wavelengths between 200 and 235 nm is unable to penetrate the skin, resulting in relatively limited skin damage [21].

### 2.2. Signaling Pathways of UV-Induced Skin Damage and Molecular Targets of Medicinal Plants

**Figure 1:**
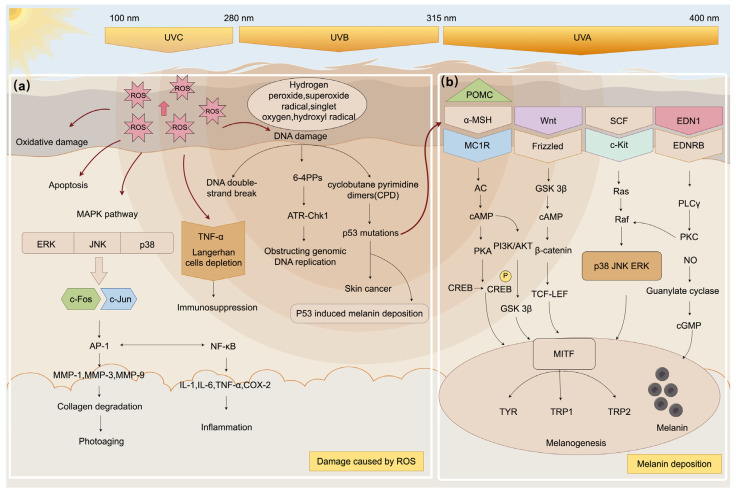
Molecular mechanisms of skin damage induced by ultraviolet radiation. (**a**) Oxidative stress and DNA damage triggered by UV. Ultraviolet (UV) radiation, encompassing UVA, UVB, and UVC, induces the production of reactive oxygen species (ROS), leading to oxidative stress, apoptosis, immunosuppression, and inflammation. ROS activate the MAPK pathway (ERK, JNK, and p38), which upregulates transcription factors c-Fos and c-Jun, promoting collagen degradation and contributing to photoaging. Additionally, UV induces DNA damage by forming cyclobutane pyrimidine dimers (CPDs) and 6-4 photoproducts, causing genomic instability, p53 mutations, and an increased risk of skin cancer. (**b**) Regulation of melanogenesis by UV-induced signaling pathways. UV radiation promotes melanin deposition through multiple signaling pathways, including α-MSH/MC1R, Wnt/β-catenin, SCF/c-Kit, EDN1/EDNRB, and NO signaling. These pathways converge on the transcription factor MITF, which orchestrates the regulation of melanogenesis.

UV radiation triggers skin DNA damage, oxidative stress, and increased inflammatory cytokines, activating various signaling pathways (Figure 1). Components such as vitamins, flavonoids, and phenolic acids in medicinal plant extracts exhibit antioxidant properties. By modulating key signaling molecules and consequently influencing the expression of downstream genes, these compounds combat UV-induced oxidative stress and mitigate the resulting damage.

#### 2.2.1. Oxidative Stress

Oxidative stress is defined as the excessive accumulation of oxygen free radicals in the body, with unremoved reactive oxygen species (ROS) leading to skin tissue damage. UV radiation induces ROS production, and prolonged exposure results in their accumulation. Persisting ROS activate the MAPK signaling pathway. The MAPK family, comprising ERK, p38, and JNK, is activated and facilitates the interaction between c-fos and c-jun, forming the AP-1 heterodimer. This process upregulates matrix metalloproteinases (MMPs) like MMP-1, MMP-3, and MMP-9, degrading the extracellular matrix and damaging dermal collagen and elastic fibers, thereby causing skin injury [22]. Furthermore, MAPK acts as an upstream regulator of the NF-κB signaling pathway. UV exposure triggers the release of inflammatory cytokines, including IL-1, IL-6, and TNF-α, intensifying the inflammatory response.

Specific components in medicinal plants help regulate intracellular balance, alleviating photoaging. For example, ginsenosides exhibit antioxidant properties by suppressing the MAPK, AP-1, and NF-κB signaling pathways, reducing MMP expression, and mitigating photoaging [23]. The methanol extract of *Malus baccata* shows remarkable effects, suppressing the expression of inflammatory mediators like MMPs, COX-2, and IL-1β in HaCaT cells and enhancing levels of skin repair proteins such as COL1A1. Phenolic and flavonoid compounds in *Houttuynia cordata* extract reduce ROS generation and suppress IL-6 and IL-8 secretion, effectively mitigating UVB-induced skin damage [24].

#### 2.2.2. DNA Damage

DNA bases contain conjugated double bonds within their aromatic rings, allowing them to absorb UV radiation, particularly in the UVB (280–320 nm) range, leading to direct DNA lesions. The damage includes the formation of cyclobutane pyrimidine dimers (CPDs) and pyrimidine(6-4)pyrimidone photoproducts (6-4PPs) as direct photochemical lesions, as well as oxidative base modifications and DNA strand breaks resulting from secondary oxidative stress [25,26]. CPD accumulation can trigger cellular stress responses, leading to increased mitochondrial ROS production and, over time, p53 mutations, which may contribute to skin carcinogenesis [27]. If left unrepaired, these DNA lesions progressively accumulate, disrupting DNA damage response pathways, impairing immune surveillance, and ultimately leading to apoptosis or carcinogenesis.

Several medicinal plant extracts mitigate apoptosis by activating DNA repair mechanisms. These pathways involve nucleotide excision repair (NER), base excision repair (BER), and homologous recombination repair. Ginsenoside Rb1 and tea polyphenols, known for their antioxidant properties, activate DNA repair pathways and avert DNA damage accumulation [28]. Additionally, resveratrol, a natural phenolic compound, eliminates excess ROS and mitigates DNA double-strand breaks [29]. Similarly, ethanol extracts of *Eclipta prostrata* efficiently neutralize free radicals and alleviate UV-induced DNA damage [30].

#### 2.2.3. Immunosuppression

Langerhans cells (LCs), key antigen-presenting cells in the epidermis, present antigens to T lymphocytes upon external stimulation, triggering immune responses [31]. UV exposure triggers the release of TNF-α (tumor necrosis factor-alpha), which induces morphological changes in LCs, hampers their migratory ability, and disrupts antigen presentation to T cells, leading to immunosuppression [32].

#### 2.2.4. Melanin Hyperpigmentation

Skin pigmentation primarily depends on the distribution and abundance of melanosomes within keratinocytes, which receive them from melanocytes through dendritic transfer. While melanin plays a crucial role in absorbing and dissipating UV radiation to protect skin cells from DNA damage, research indicates that UV exposure dysregulates multiple melanogenesis pathways, often leading to excessive pigmentation [33].

a. α-MSH/MC1R Pathway

UV-induced DNA damage can activate p53, stimulating the synthesis of the α-melanocyte-stimulating hormone (α-MSH). Upon binding to the melanocortin 1 receptor (MC1R) on melanocytes, α-MSH activates adenylate cyclase (AC), leading to the production of the second messenger cyclic adenosine monophosphate (cAMP). cAMP binds to the regulatory subunits of protein kinase A (PKA), releasing its catalytic subunits, which then activate PKA. Activated PKA phosphorylates the cAMP response element-binding protein (CREB), a transcription factor that regulates microphthalmia-associated transcription factor (MITF), the key regulator of melanin biosynthesis. Medicinal plant extracts can mitigate α-MSH-induced oxidative stress and hyperpigmentation by elevating intracellular glutathione (GSH) levels [34].

b. Wnt/Frizzled Pathway

The Wnt signaling pathway is critical for the proliferation and differentiation of melanocyte stem cells (McSCs). Research indicates that UVB exposure stimulates keratinocytes to release Wnt7A, which is significantly upregulated. Wnt7A binds to frizzled receptors on McSCs in hair follicles, promoting nuclear translocation of β-catenin and the rapid differentiation of McSCs into melanocytes. Subsequently, these newly differentiated melanocytes migrate from the hair follicles to the epidermis, undergo proliferation, and initiate melanin synthesis [35]. The UVB-induced Wnt/β-catenin pathway activation promotes melanocyte formation and migration while also activating MITF, a key regulator of pigmentation. MITF further upregulates the expression of melanin-synthesizing enzymes like tyrosinase (TYR), TRP1, and TRP2, intensifying UVB-induced hyperpigmentation and photoaging.

c. SCF/c-Kit Pathway

UVB exposure induces the expression of the stem cell factor (SCF) in epidermal cells. SCF interacts with the c-Kit receptor, activating Ras protein, which subsequently activates Raf and triggers the MAPK signaling pathway [36]. This cascade regulates MITF expression and facilitates melanin synthesis. During this pathway, p38 activation increases CREB transcriptional activity, stimulating melanin synthesis. However, phosphorylation of ERK and JNK can suppress MITF transcription, thereby serving to inhibit melanin production under specific conditions [37].

d. EDN1/EDNRB Pathway

UVB irradiation stimulates the secretion of endothelin 1 (EDN1) by keratinocytes [38]. Upon binding to its receptor, endothelin receptor B (EDNRB), EDN1 activates phospholipase Cγ (PLCγ), generating inositol trisphosphate (IP3) and triggering the release of intracellular calcium ions. This subsequently activates protein kinase C (PKC), which phosphorylates Raf, initiating the MAPK signaling cascade. This cascade modulates the activity of MITF and its downstream enzymes, ultimately promoting melanin synthesis [39].

e. NO Signaling Pathway

UV irradiation stimulates the production of nitric oxide (NO) in skin cells. NO activates soluble guanylate cyclase (sGC), which catalyzes the conversion of guanosine triphosphate (GTP) to cyclic guanosine monophosphate (cGMP), further upregulating MITF expression, thus promoting melanin production [40].

## 3. Therapeutic Potential of Medicinal Plants in UV-Induced Skin Damage

Prolonged exposure to UVA and UVB radiation can cause various types of skin damage, including sunburn, skin ulcers, photoaging, and even skin cancer. Each type presents distinct symptoms and therapeutic needs, and medicinal plants have shown significant potential in alleviating these skin issues. Sunburn, characterized by erythema and pruritus, represents acute epidermal damage and is primarily treated with medicinal plants possessing anti-inflammatory and antipruritic properties. Skin ulcers involve deeper dermal damage, often with increased exudate and susceptibility to infection, requiring plants with antibacterial, anti-inflammatory, and wound-healing properties. Photoaging, caused by UV-induced ROS generation, manifests as skin dryness and wrinkles, for which antioxidant plants are key therapeutic agents. Skin cancer, the most severe consequence of cumulative UV damage, may be prevented or treated with medicinal plants exhibiting anticancer activity and the ability to inhibit cancer cell growth.

The 18 plants featured in this review were selected based on their established use in traditional medicine and proven efficacy in treating UV-induced skin damage. Their active compounds have demonstrated antioxidant, anti-inflammatory, and DNA repair properties, with promising results in preclinical studies (Table 1).

### 3.1. Salvia miltiorrhiza

*Salvia miltiorrhiza* (Danshen), a dicotyledonous plant of the Lamiaceae family, is renowned for its therapeutic potential and is rich in active compounds such as Tanshinone I, Tanshinone II, danshensu (DSU), and salvianolic acid B (SAB). These compounds exhibit antioxidant, anti-inflammatory, antifibrotic, and neuroprotective effects, contributing to their broad medicinal applications [41]. The primary medicinal parts of *S. miltiorrhiza* are its leaves and roots. Leaf extracts, abundant in phenolic compounds, act as natural antioxidants that effectively neutralize UV-induced reactive oxygen species (ROS). Root extracts, on the other hand, reduce melanin accumulation and mitigate allergic skin responses [42,43].

Recent studies have revealed the potential of DSU and SAB in regulating melanin synthesis through tyrosinase inhibition. DSU exhibits a half-maximal inhibitory concentration (IC50) of 44.1 ± 1.1 mM, indicating a potent inhibitory effect. In α-MSH-stimulated B16 melanoma cells, both DSU and SAB effectively reduced intracellular melanin levels. Specifically, 2 mM DSU decreased α-MSH-induced melanin content by 15%, while 0.5 mM SAB reduced it by approximately 20%, demonstrating their ability to inhibit tyrosinase activity and suppress melanogenesis [44].

In addition to melanogenesis regulation, *S. miltiorrhiza* extracts also show promise in mitigating UV-induced skin aging. Using UVA (15 J/cm^2^) and UVB (200 mJ/cm^2^) to simulate photoaging, studies on human keratinocyte (HaCaT) and human fibroblast (HFF-1) cell lines revealed that pre-treatment with 0.1 μM cryptotanshinone (CTS) significantly reduced the proportion of senescence-associated β-galactosidase (SA-β-gal)-positive cells and lowered the mRNA levels of senescence-associated secretory phenotype (SASP) factors, including IL-6, IL-8, MMP-1, and MMP-3. Mechanistically, CTS was shown to activate the Nrf2 signaling pathway, which attenuates ROS generation and DNA damage. Additionally, it activated the AMPK/SIRT1/PGC-1α pathway, reducing mitochondrial dysfunction and promoting mitochondrial biogenesis [45].

Moreover, the wound-healing potential of *S. miltiorrhiza* extract is highly concentration-dependent. A 5% extract exhibits pronounced wound-healing effects by leveraging its strong antibacterial and antioxidant properties to promote angiogenesis, thereby facilitating tissue regeneration [46]. Collectively, these findings highlight the multifaceted roles of *S. miltiorrhiza* and its active compounds in addressing UV-induced skin damage through antioxidative, anti-melanogenic, and anti-photoaging mechanisms.

### 3.2. Panax notoginseng

*Panax notoginseng* (Sanqi), a member of the Araliaceae family, is highly regarded for its medicinal properties, primarily derived from its roots and rhizomes. Key active ingredients include notoginsenosides and ginsenosides. Fresh *P. notoginseng* exhibits notable hemostatic, anticoagulant, and anti-inflammatory activities, while steamed *P. notoginseng* demonstrates antioxidant and hematopoietic properties [47]. In treating skin injuries, notoginsenosides suppress the proliferation of NIH-3T3 fibroblasts, effectively reducing scar formation. They also display anti-inflammatory, antioxidant, and antipruritic effects, significantly enhancing wound healing. Incorporating low concentrations of notoginsenosides into hydrogels has been shown to enhance biocompatibility, optimize wound conditions, strengthen antibacterial activity, and accelerate wound closure [48,49].

Recent studies have highlighted the therapeutic potential of *P. notoginseng* flower extract in mitigating UV-induced skin damage. At a concentration of 200 μg/mL, *P. notoginseng* flower saponins (PNFSs) exhibit strong anti-inflammatory activity by upregulating the expression of antimicrobial peptide LL-37 and suppressing UVB-induced increases in PGE-2, IL-1β, and TNF-α levels in HaCaT cells [50].

In vitro experiments further revealed the efficacy of *P. notoginseng*-derived protopanaxtriol saponins (PTSs) in regulating melanogenesis. Pre-treatment of B16 melanoma cells with 0.1 mg/mL PTS for 1 h, followed by exposure to 20 nM α-MSH, significantly inhibited tyrosinase activity. Among the tested ginsenosides, Rg1 and Re demonstrated the most potent inhibitory effects on melanin synthesis.

In vivo studies employed a PTS ethosome system to enhance dermal delivery. The ethosome system, characterized by high deformability and fluidity, effectively penetrated the stratum corneum, significantly improving drug permeation compared to traditional methods. Topical application of a 10 mg/mL PTS ethosome gel to UVB-irradiated dorsal skin of C57BL/6 mice markedly suppressed melanin production, mitigating UVB-induced hyperpigmentation [51].

Collectively, these findings underscore the potential of *P. notoginseng*, particularly its flower-derived saponins and advanced delivery systems, in addressing UV-induced skin damage through anti-inflammatory, anti-melanogenic, and antioxidant mechanisms.

### 3.3. Astragalus membranaceus

*Astragalus membranaceus* (Huangqi), a renowned traditional medicinal herb, has garnered significant attention for its active compounds’ ability to counteract UV-induced skin damage. Polysaccharide APS2-1, derived from *A. membranaceus*, has demonstrated efficacy in promoting fibroblast proliferation and accelerating wound healing in vitro [52]. Additionally, *A. membranaceus* contains saponins such as astragaloside IV (AS-IV) and cycloastragenol I, which enhance fibroblast proliferation and migration, thereby facilitating skin repair [53]. Compounds like astragalus gum have been reported to promote wound contraction and repair effectively [54]. During photoaging, UVB radiation activates NF-κB, resulting in the upregulation of matrix metalloproteinase MMP-1, which degrades type I collagen. Root extract of *A. membranaceus* has been shown to suppress NF-κB activity, preserving collagen integrity in fibroblasts [55].

Recent studies have further elucidated the molecular mechanisms underlying the photoprotective effects of *A. membranaceus* polysaccharide (AP) and AS-IV. MTT assays have confirmed that AP exhibits no cytotoxicity toward HaCaT cells at concentrations ranging from 50 to 600 μg/mL. At 500 μg/mL, AP significantly reduced ROS expression in HaCaT cells exposed to UVA (30 mJ/cm^2^). Furthermore, AP enhances mitochondrial function by improving the activity of complex I and complex II, thereby protecting HaCaT cells against ATP depletion and mitochondrial membrane potential (MMP) reduction caused by UVA exposure. These effects collectively safeguard skin from photodamage [56].

AS-IV, another key component of *A. membranaceus*, offers additional protection against UVB-induced inflammation and oxidative stress by modulating the TLR4 signaling pathway in keratinocytes. At a concentration of 50 μM, AS-IV exhibits no cytotoxic effects on UVB-irradiated HaCaT cells while effectively reducing intracellular ROS levels and malondialdehyde (MDA) content. Concurrently, AS-IV increases superoxide dismutase (SOD) activity, counteracting the negative effects of UVB radiation [57].

In summary, *A. membranaceus* employs a multifaceted approach to mitigate UV-induced skin damage, leveraging its polysaccharides and saponins to combat oxidative stress, inflammation, and collagen degradation. These findings highlight its potential as a valuable therapeutic agent for skin protection and repair.

### 3.4. Andrographis paniculata

*Andrographis paniculata* (green chiretta), a medicinal plant in the Acanthaceae family, is renowned for its active compound andrographolide, which exhibits potent anti-inflammatory properties. This compound is particularly effective in addressing skin inflammation and wounds caused by bacterial or viral infections [58,59]. However, its poor water solubility and limited bioavailability present challenges, which can be addressed by synthesizing derivatives or developing innovative delivery systems [60]. Both polar and non-polar extracts of *A. paniculata* demonstrate strong antibacterial activity against Gram-positive and Gram-negative bacteria, reducing wound infections and minimizing the risk of resistance development [60]. Notably, a 10% concentration of *A. paniculata* extract significantly enhances fibroblast proliferation and angiogenesis, proving more effective than its 5% counterpart [61]. The leaf extract, rich in flavonoids, tannins, and alkaloids, contains UV-absorbing compounds that provide photoprotective effects, particularly within the 290–400 nm range [62]. Additionally, both the methanol extract of *A. paniculata* leaves and andrographolide effectively scavenge UV-induced reactive oxygen species (ROS), thereby reducing oxidative stress-induced inflammation [63].

Recent studies have further highlighted the photoprotective potential of andrographolide (ADP) in vivo. In a mouse model exposed to 180 mJ/cm^2^ UVB radiation, the oxidative stress induced by UVB led to a significant reduction in antioxidant enzyme activity and glutathione (GSH) levels, alongside an increase in thiobarbituric acid reactive substances (TBARSs), a marker of lipid peroxidation. Pre-treatment with ADP at a dose of 3.6 mg/kg body weight (b.wt) successfully restored antioxidant enzyme activity and GSH content while reducing TBARS levels, demonstrating its antioxidant efficacy. Additionally, ADP downregulated CXCL1 expression and inhibited the release of pro-inflammatory cytokines such as TNF-α, IL-6, and IL-1β. Remarkably, this protective effect persisted for up to 10 days post-irradiation, indicating the long-term benefits of ADP in shielding the skin from UVB-induced damage [64].

In conclusion, *A. paniculata* and its active compound andrographolide exhibit multifaceted protective effects against UVB-induced skin damage, including antioxidant activity, anti-inflammatory effects, and photoprotection. These properties underscore its potential as a therapeutic agent for mitigating oxidative stress and inflammation in UV-damaged skin.

### 3.5. Paeonia lactiflora

*Paeonia lactiflora* (Chinese peony), a member of the Ranunculaceae family, is widely used in traditional medicine for its pharmacological properties. Its bioactive constituents include total paeony glycosides, such as paeoniflorin, oxypaeoniflorin, and hydroxy-paeoniflorin, which have demonstrated antibacterial, anti-inflammatory, antioxidant, and neuroprotective effects [65,66,67,68]. Among these, paeoniflorin has been identified as a key compound with potent antioxidant properties, primarily through its regulation of the ROS/p38/p53 pathway, making it a promising candidate for alleviating UVB-induced skin damage [69]. Additionally, acidic polysaccharide RPAPS from *P. lactiflora* exhibits strong DPPH radical scavenging activity, effectively counteracting UV-induced oxidative stress [70].

Beyond its antioxidant capacity, *P. lactiflora* extracts also play a significant role in regulating melanogenesis and mitigating UV-induced skin inflammation. In an in vitro study using B16-F10 melanoma cells, α-MSH was found to induce a dose-dependent increase in melanin synthesis, tyrosinase activity, and the expression of key melanogenic proteins, including TRP-1, TRP-2, and MITF. However, treatment with 10 µM paeoniflorin effectively inhibited α-MSH-induced melanogenesis by suppressing CREB activation, leading to reduced tyrosinase activity and melanin production [71]. These findings suggest that paeoniflorin could be a promising depigmenting agent for hyperpigmentation disorders caused by UV exposure.

Another key bioactive compound from *P. lactiflora* is paeonol, which has been extensively studied for its anti-inflammatory properties. A study investigated the effects of paeonol in UV-induced skin inflammation using human HaCaT keratinocytes and JB6 Cl41 mouse epidermal cells as models. Upon exposure to 20 kJ/m^2^ solar ultraviolet (SUV) radiation, cells exhibited epidermal hyperplasia, inflammatory cell infiltration, and intercellular edema, hallmarks of UV-induced inflammation. Treatment with 400 µM paeonol significantly attenuated these effects by inhibiting the phosphorylation of MSK1 (Mitogen- and stress-activated kinase 1) and histone H2AX while blocking TOPK (T-LAK cell-originated protein kinase) activity. This inhibition led to the downregulation of key inflammatory mediators in the MAPK pathway, including phosphorylated p38 and JNK, resulting in reduced secretion of pro-inflammatory cytokines such as IL-6 and TNF-α [72].

Collectively, these findings highlight the multifunctional potential of *P. lactiflora* extracts in mitigating UV-induced skin damage. Paeoniflorin contributes to photoprotection by modulating oxidative stress and inhibiting melanogenesis, while paeonol exhibits potent anti-inflammatory effects by regulating inflammatory signaling pathways. These mechanisms support the therapeutic application of *P. lactiflora* in preventing UV-induced skin aging and hyperpigmentation.

### 3.6. Panax ginseng

*Panax ginseng* (Ginseng), a perennial herb from the Araliaceae family, has long been recognized for its extensive pharmacological benefits. Its bioactive constituents, primarily ginsenosides and ginseng oligosaccharides (GSOs), contribute to skin protection and repair [73]. Additionally, enzyme-modified ginseng leaf extracts using Ultraflo L have been shown to suppress matrix metalloproteinase (MMP) expression, particularly MMP-2, MMP-3, and MMP-13, which play key roles in extracellular matrix degradation and photoaging [74].

One of the major bioactive saponins in *P. ginseng* is Compound K (CK), which exhibits potent anti-photoaging effects. In an in vitro study, NIH3T3 fibroblasts pre-treated with 10 µM CK prior to UVB irradiation exhibited significantly reduced levels of MMP-1 and COX-2, key enzymes involved in collagen degradation and inflammation. Furthermore, CK treatment restored type I collagen expression, alleviated UV-induced skin dryness, and effectively counteracted photoaging [75].

GSO, another key component extracted from *P. ginseng* roots, has demonstrated promising effects in both in vivo and in vitro models of UV-induced skin damage. In BALB/c nude mice, topical application of GSOs at a concentration of 20 µg/mL to the dorsal skin significantly upregulated both mRNA and protein levels of filaggrin (FLG), involucrin (IVL), and aquaporin-3 (AQP3), thereby improving skin hydration and enhancing desquamation-associated proteins. Additionally, in human keratinocyte cultures HaCaT, GSOs enhanced the expression of serine protease inhibitor Kazal type-5 (SPINK5) and desmoglein 1 (DSG1) while downregulating trypsin-like kallikrein-related peptidase 5 (KLK5) and chymotrypsin-like KLK7, which are implicated in skin barrier disruption, indicating their strong potential for barrier repair [76].

Further evidence supports the protective role of *P. ginseng* extract in preventing UVB-induced apoptosis and extracellular matrix degradation. Treatment with 40 µg/mL *P. ginseng* extract upregulated the expression of vacuole membrane protein 1 (VMP1), a key regulator of endoplasmic reticulum stress, thereby reducing UVB-induced apoptosis. In BALB/c mice, oral administration of *P. ginseng* extract at 4 mg/g body weight significantly reduced tissue damage and apoptosis while suppressing MMP-1, MMP-2, and MMP-9 expression, which are closely linked to skin aging [77]. Additionally, topical application of *P. ginseng* extract at a dose of 2 mg/cm^2^ to UVB-irradiated mouse skin prevented the UVB-induced downregulation of FLG, transglutaminase-1 (TGM1), and hyaluronan synthases (HAS1, HAS2, and HAS3), genes essential for skin hydration and repair. Concurrently, it suppressed MMP-1, MMP-2, and MMP-9 expression, thereby preventing UVB-induced collagen degradation and skin aging [78].

Taken together, these findings highlight the multifaceted protective effects of *P. ginseng* against UV-induced skin damage. By modulating oxidative stress, inflammatory signaling, and extracellular matrix remodeling, *P. ginseng* extracts hold significant promise in mitigating photoaging, enhancing skin hydration, and reinforcing the epidermal barrier.

### 3.7. Scutellaria baicalensis

*Scutellaria baicalensis* (Chinese skullcap), a perennial herb of the Lamiaceae family, is primarily valued for its medicinal roots, which are rich in flavonoids with diverse pharmacological properties. The roots of *S. baicalensis* contain over 30 flavonoids, with baicalin and baicalein being the major components. These bioactive constituents exhibit potent antioxidant, anticancer, and anti-inflammatory effects, making *S. baicalensis* a promising candidate for skin protection [79,80,81].

Research has highlighted the role of flavonoids such as baicalein and wogonin in mitigating UV-induced skin damage. In vivo studies demonstrated that oral administration of 50 mg/kg baicalein or 50 mg/kg wogonin twice daily for 14 days significantly alleviated UVB-induced epidermal and dermal thickening in mice. Both flavonoids inhibited the expression of MMP-9 and vascular endothelial growth factor (VEGF), contributing to reduced extracellular matrix degradation and neovascularization. In vitro experiments revealed that baicalein (100 μM) suppresses NF-κB/p65 expression, while wogonin (100 μM) reduces HIF-1α levels, indicating that their photoprotective effects are mediated through distinct molecular pathways. However, both compounds were found to effectively downregulate UVB-induced COX-2 expression, highlighting their shared anti-inflammatory mechanism [82].

Collectively, these findings underscore the potential of *S. baicalensis* flavonoids as promising agents for protecting against UV-induced skin damage. By modulating inflammatory and oxidative stress pathways, these bioactive compounds offer a multifaceted approach to mitigating photoaging and maintaining skin integrity.

### 3.8. Rhodiola rosea

*Rhodiola rosea* (golden root), a perennial herb of the Crassulaceae family, is widely recognized for its skin-protective properties. The primary bioactive compounds in *R. rosea* are rosavin and salidroside, which exhibit potent antioxidant and anti-inflammatory activities. Studies have identified 27 active constituents from *R. rosea* crude extract capable of penetrating the epidermis and reaching the basal and dermal layers. These compounds inhibit MMP-2 and collagenase activity, thereby reducing elastin degradation and protecting against UV-induced skin damage [83,84]. Among these constituents, kaempferol has been highlighted for its strong antioxidant and photoprotective effects, significantly suppressing pro-inflammatory cytokines such as IL-6 and TNF-α [56]. Additionally, *R. rosea* extract fermented with *Lactobacillus plantarum* markedly activates the Nrf2/Keap1 pathway, thereby enhancing fibroblast antioxidant defenses and mitigating oxidative stress caused by UVA exposure [85].

Recent investigations have further elucidated the molecular mechanisms underlying *R. rosea*’s photoprotective effects. Pre-treatment with 100 μM salidroside was shown to upregulate the expression of Beclin-1 and ATG7 while downregulating P62, indicating an autophagy-dependent mechanism mediated by SIRT1 signaling. This mechanism plays a crucial role in mitigating UVB-induced cellular damage [86]. Furthermore, 50 μg/mL rosavin effectively alleviated UVA- and UVB-induced keratinocyte photoaging, as evidenced by a reduction in senescent cell populations, increased telomerase activity, and decreased DNA damage. Notably, a potential synergistic effect between salidroside and rosavin has been observed, wherein their combined application exerts greater protective effects than either compound alone [87].

These findings underscore the therapeutic potential of *R. rosea* in combating UV-induced skin damage. Through its ability to modulate autophagy, enhance cellular antioxidant defenses, and prevent collagen degradation, *R. rosea* presents a promising strategy for mitigating photoaging and preserving skin health.

### 3.9. Tripterygium wilfordii

*Tripterygium wilfordii* (Thunder god vine), a member of the Celastraceae family, has long been utilized in traditional medicine for its potent anti-inflammatory, antitumor, and immunosuppressive properties. Its bioactive constituents include triptolide, celastrol, and alkaloids, though their therapeutic use is often limited by toxicity concerns [88]. One of its refined extracts, the multi-glycoside of *T. wilfordii* (GTW), consists of trace diterpenes, minor alkaloids, and pentacyclic triterpenoids. Compared to triptolide and celastrol, GTW exhibits reduced toxicity while retaining significant immunomodulatory effects. Studies have shown that GTW alleviates imiquimod (IMQ)-induced psoriasis-like lesions in mice by inhibiting STAT3 phosphorylation and suppressing Th17 cell activity [89]. Additionally, GTW has been found to regulate immune responses and keratinocyte proliferation, while downregulating pro-inflammatory cytokines IL-17A and IL-23, thereby promoting skin repair and reducing inflammation [90,91].

Beyond its immunomodulatory effects, *T. wilfordii* also demonstrates potential in mitigating UV-induced skin damage. Celastrol, a triterpenoid extracted from *T. wilfordii*, has been shown to exhibit antiproliferative effects against melanoma cells. In vitro studies indicate that 10 μM celastrol significantly inhibits melanoma cell viability and colony formation. Its mechanism of action involves the suppression of the PI3K/AKT/mTOR signaling pathway and the downregulation of HIF-1α mRNA expression, leading to reduced melanoma cell proliferation and migration [92]. While these findings highlight the potential of celastrol in targeting aberrant skin cell proliferation, further studies are needed to establish its direct role in UV-induced skin pathologies.

Overall, *T. wilfordii* and its bioactive derivatives exhibit multifaceted therapeutic potential in skin health. Through its dual actions in modulating immune responses and targeting hyperproliferative skin cells, *T. wilfordii* represents a promising candidate for further investigation in the prevention and treatment of UV-associated skin disorders.

### 3.10. Paeonia suffruticosa

*Paeonia suffruticosa* (Moutan peony), a deciduous shrub of the Ranunculaceae family, has long been used in traditional medicine for its anti-inflammatory and antioxidant properties. Its medicinal value primarily derives from its dried root bark (Danpi), which contains paeonol as the major bioactive compound [93]. Paeonol has been shown to mitigate UV-induced skin damage by modulating key inflammatory and oxidative stress pathways. Specifically, it inhibits T-LAK cell-originated protein kinase (TOPK) activity within the MAPK pathway, thereby reducing the phosphorylation of downstream effectors [72].

Beyond its anti-inflammatory effects, paeonol also attenuates UVB-induced photoaging by protecting skin cells from oxidative stress and extracellular matrix degradation. In vitro studies utilizing HaCaT keratinocytes revealed that 10 μg/mL paeonol significantly inhibited the UVB-induced phosphorylation of MAPK components, including ERK, JNK, and p38, thereby suppressing AP-1 activation. Consequently, this led to a reduction in MMP-1 expression and an increase in procollagen type I synthesis, suggesting a protective role against UVB-induced extracellular matrix degradation [94]. Furthermore, paeonol activated the endogenous antioxidant defense system through the Nrf2/antioxidant response element (ARE) pathway. Under oxidative stress, Nrf2 translocates into the nucleus, binding to ARE sequences to promote the expression of antioxidant enzymes such as NAD(P)H:quinone oxidoreductase 1 (NQO-1) and heme oxygenase-1 (HO-1). Paeonol treatment further enhanced Nrf2 nuclear translocation, restoring dihydrolipoyl dehydrogenase (DLD) levels, which were downregulated upon UVB exposure. This suggests that paeonol protects against UVB-induced oxidative stress by activating the DLD/Nrf2/ARE axis [94].

In vivo experiments using hairless mice subjected to chronic UVB irradiation demonstrated that topical application of 1% *P. suffruticosa* roots extract or 0.1% paeonol significantly reduced epidermal hyperplasia and dermal collagen degradation. The extract suppressed UVB-induced MMP-1 expression while enhancing procollagen type I levels, thereby attenuating wrinkle formation and improving skin elasticity [94]. These findings indicate that *P. suffruticosa* and its bioactive component paeonol hold promise as effective botanical agents for protecting the skin against UVB-induced photoaging through dual mechanisms: inhibiting the MAPK/AP-1 pathway and activating the DLD/Nrf2/ARE pathway.

### 3.11. Centella asiatica

*Centella asiatica* (Gotu kola), belonging to the Apiaceae family, possesses notable antibacterial activity, accelerates wound healing, and minimizes scar formation [95]. Extracts from the roots and leaves of *C. asiatica* are rich in asiaticoside, demonstrating high antioxidant activity that neutralizes free radicals and shields skin from oxidative damage. Studies indicate that hexane, ethyl acetate, methanol, and low-concentration aqueous extracts of *C. asiatica* effectively stimulate collagen synthesis and angiogenesis, thereby accelerating wound healing [96,97].

Recent studies have further explored the photoprotective effects of asiatic acid, a major triterpenoid derived from *C. asiatica*, particularly when formulated with glucosamine salt to enhance bioavailability. Asiatic acid glucosamine salt (AAGS) exhibits self-assembling properties, forming hydrogels suitable for topical application. In vivo experiments demonstrate that the application of 60 mg/mL AAGS gel to the dorsal skin of nude mice 30 min before UVB exposure significantly mitigates UVB-induced wrinkle formation, increases skin thickness, elevates collagen content, and reduces the expression of matrix metalloproteinases (MMPs), nearly restoring skin integrity to normal levels. Moreover, AAGS effectively prevents UVB-induced oxidative stress by reducing intracellular ROS levels in human dermal fibroblasts (HDFs) and inhibiting cellular senescence, as evidenced by a decrease in senescence-associated β-galactosidase (SA-β-Gal) positive cells [98].

In addition to AAGS, a standardized extract of *C. asiatica* known as titrated extract of *Centella asiatica* (TECA) has been shown to provide strong protection against UVB-induced damage in human dermal fibroblasts (NHDFs). TECA contains a mixture of asiatic acid, madecassic acid, asiaticoside, and madecassoside, and has been widely used for its wound-healing and anti-aging properties. In vitro studies demonstrate that pre-treatment with 5 µg/mL TECA significantly increases cell viability following UVB irradiation, while 25–50 µg/mL TECA markedly restores UVB-induced loss of cell survival. Mechanistically, TECA exerts its protective effects by altering miRNA expression profiles in NHDFs, leading to the upregulation of miRNAs associated with cell proliferation and the downregulation of those linked to apoptosis. Bioinformatic analysis suggests that TECA-mediated miRNA regulation primarily influences pathways involving MAPK, Ras, and small GTPase signaling, ultimately enhancing fibroblast resilience against UVB-induced oxidative stress [99].

Collectively, these findings highlight the potential of *C. asiatica* extracts, particularly AAGS and TECA, as effective agents for mitigating UVB-induced skin damage through mechanisms that involve oxidative stress reduction, collagen preservation, and cellular signaling modulation.

### 3.12. Arctium lappa

*Arctium lappa* (Greater burdock), a member of the Asteraceae family, is rich in arctiin, a lignan glycoside found in its leaves and roots. Arctiin exhibits potent anti-inflammatory and antiviral properties and has been traditionally used in the treatment of burns, gastric ulcers, and other inflammatory disorders [100].

Studies have highlighted the protective role of arctiin against UV-induced skin damage, particularly in human dermal fibroblasts (NHDFs). UVB exposure leads to cumulative DNA damage, oxidative stress, and apoptosis in NHDFs, ultimately impairing skin integrity. However, pre-treatment with 10 µM arctiin for six hours significantly mitigates UVB-induced apoptosis and promotes DNA repair, restoring fibroblast viability [101]. Mechanistically, arctiin exerts its protective effects against UV-induced damage by modulating microRNA (miRNA) expression and regulating key signaling pathways. In HaCaT keratinocytes, arctiin significantly alters the miRNA expression profile, notably upregulating miR-205-3p while downregulating miR-3652, miR-513a-5p and miR-1290, which are associated with MAPK signaling pathway. These miRNA shifts help counteract UV-induced oxidative stress and apoptosis, ultimately promoting cell survival and skin regeneration [102]. Additionally, arctiin downregulates miR-378b, which negatively regulates sirtuin-6 (SIRT6), a key factor in collagen type 1α 1 chain (COL1A1) synthesis. By suppressing miR-378b, arctiin increases SIRT6 expression, leading to enhanced collagen preservation, thereby mitigating UV-induced photoaging and maintaining skin elasticity [103].

These findings underscore the potential of arctiin as a protective agent against UVB-induced skin damage, with its effects primarily mediated through DNA repair enhancement, miRNA modulation, and collagen preservation.

### 3.13. Aloe vera

*Aloe vera* (Aloe), a herbaceous plant in the Asphodelaceae family, is rich in aloesin, a bioactive compound with significant potential in treating UV-induced burns and preventing UVB-induced immunosuppression [104]. Aloesin activates the MAPK/Rho and TGF-β signaling pathways, facilitating wound healing and tissue regeneration [105]. Uncontrolled UVB-induced inflammation is a critical factor contributing to photoaging and skin tumorigenesis, with cyclooxygenase-2 (COX-2) playing a central role. Studies indicate that Aloe suppresses UVB-induced COX-2 expression, thereby blocking inflammatory signaling cascades and mitigating UV-induced skin inflammation [106].

Furthermore, the amino acids and organic acids in Aloe exhibit tyrosinase inhibitory activity, reducing melanin production and alleviating UV-induced hyperpigmentation. Recent studies highlight the enhanced skin-brightening potential of *Lactobacillus plantarum* BN41-fermented Aloe leaf extract (BF). The fermentation process significantly increases the bioavailability of active compounds, leading to potent antioxidant properties and melanin synthesis inhibition. Mechanistically, BF downregulates tyrosinase-related protein-1 (TYRP-1) and TYRP-2, crucial enzymes in melanin biosynthesis, thereby reducing pigmentation in UV-exposed skin. Among tested concentrations, 0.3% (w/v) BF demonstrated the strongest skin-lightening effects, optimizing both antioxidant activity and melanogenesis suppression [107]. These findings further validate Aloe as a promising agent for UV-induced skin damage prevention and cosmetic dermatology applications.

### 3.14. Sophora flavescens

*Sophora flavescens* (Shrubby sophora), a member of the Fabaceae family, is well known for its alkaloid-rich composition, including matrine and oxymatrine, which exert potent anti-inflammatory effects. These alkaloids suppress IL-8 and TNF-α expression, inhibit inflammatory cell migration and activation, and alleviate skin redness, swelling, and pain [108]. Moreover, *S. flavescens* displays broad-spectrum antibacterial properties, effectively inhibiting pathogens such as *Staphylococcus aureus*, *Staphylococcus epidermidis*, and *Propionibacterium acnes*. When combined with azelaic acid, its skin penetration and therapeutic efficacy are significantly enhanced [109]. Additionally, *S. flavescens* exhibits antipruritic effects by modulating calcium channel activity, which influences neurotransmitter synthesis and release, thereby reducing itching sensations [110]. However, due to its potential toxicity, long-term or high-dose usage should be cautiously administered.

Beyond its anti-inflammatory and antibacterial effects, flavonoids such as Kushenol C and Kuraridin, isolated from the *S. flavescens* root, have demonstrated protective effects against UV-induced skin damage. Oral administration of 10 mg/kg Kushenol C to mice subjected to 120 mJ/cm^2^ UVB radiation for 14 consecutive days significantly reduced malondialdehyde (MDA) levels, an oxidative stress marker, while restoring the activity of glutathione (GSH), superoxide dismutase (SOD), and catalase (CAT) in the skin. Furthermore, Kushenol C effectively inhibited mast cell infiltration and alleviated epidermal thickening, thereby preventing UV-induced skin inflammation [111]. Kuraridin, another prenylated flavonoid from *S. flavescens*, has demonstrated exceptional anti-melanogenic properties through its inhibition of tyrosinase activity. In α-MSH-induced B16-F10 melanoma cells, 5 µM Kuraridin significantly reduced intracellular and extracellular melanin content by directly suppressing tyrosinase activity. Notably, Kuraridin strongly downregulated c-KIT, a receptor tyrosine kinase essential for tyrosinase activation, and inhibited ERK1/2 phosphorylation, leading to decreased MITF (Microphthalmia-associated transcription factor) expression—a master regulator of melanogenesis [112].

These findings reinforce the therapeutic versatility of *S. flavescens* in UV-induced skin damage, demonstrating antioxidant, anti-inflammatory, and depigmenting effects, thereby supporting its potential for both clinical dermatology and cosmeceutical applications.

### 3.15. Saussurea involucrata

*Saussurea* (Snow lotus), a member of the Asteraceae family, is widely recognized for its potent antioxidant properties, which contribute to its protective effects against UV-induced oxidative stress. Studies using UV-treated murine melanoma cells B16-F10 revealed that *S. involucrata* herba extract (SIH) significantly reduces ROS accumulation in a dose-dependent manner while upregulating the transcriptional activity of antioxidant response elements (AREs). SIH promotes PI3K/Akt phosphorylation, enhancing the nuclear translocation of Nrf2 and increasing the expression of detoxifying enzymes such as NQO1 and GCLM [113].

Recent studies have further demonstrated the photoprotective and anti-aging effects of *Saussurea* extracts in UV-exposed models. The ethanol extract of *Saussurea laniceps* (ESL) has been shown to enhance systemic antioxidant defenses when administered orally. In an in vivo study, mice exposed to UVA (142.50 J/cm^2^) and UVB (20.52 J/cm^2^) were treated with 360 mg/kg ESL, which significantly increased the activity of superoxide dismutase (SOD), glutathione peroxidase (GSH-PX), and hydroxyproline (HYP). These enzymatic improvements correlated with visible reductions in UV-induced skin damage, including wrinkles, erythema, and lichenification, highlighting the systemic photoprotective potential of *Saussurea* [114].

In addition to its systemic effects, *Saussurea involucrata* polysaccharide (SIP) has shown promise as a topical agent for UVB-induced skin damage. The 0.5% SIP hydrogel, applied to UVB-irradiated C57BL/6 mice, significantly reduced UVB-induced xerosis, scaling, and erythema. On a molecular level, SIP treatment decreased the accumulation of γ-H2AX and cyclobutane pyrimidine dimers (CPDs), both key markers of UV-induced DNA damage, and suppressed inflammatory responses by reducing IL-1β and TNF-α mRNA expression. These findings suggest that SIP mitigates UV-induced DNA damage and inflammatory responses by activating the PPAR-α signaling pathway, thereby preserving skin integrity and reducing photodamage [115].

Together, these findings highlight the therapeutic potential of *Saussurea* extracts in counteracting UV-induced skin damage through multiple mechanisms, including antioxidant enzyme activation, collagen preservation, inflammatory suppression, and DNA repair.

### 3.16. Houttuynia cordata

*Houttuynia cordata* (Fish mint), a member of the Saururaceae family, has been widely studied for its bioactive compounds, particularly quercitrin and hyperoside, which are abundant in its ethyl acetate extract fraction (HC-EA). These flavonoids exhibit potent antioxidant and anti-inflammatory properties, making them valuable in protecting skin from UV-induced damage. Compared to aqueous extracts, *H. cordata* extracts fermented with *Aureobasidium pullulans* show significantly increased levels of active compounds, enhancing their bioactivity and therapeutic potential [116,117].

In vitro studies have demonstrated that HC-EA effectively protects both human keratinocytes (HaCaT) and human dermal fibroblasts (HDFs) from UVB-induced oxidative stress and inflammation, though the underlying mechanisms differ between these cell types. In HaCaT cells, 50 µg/mL HC-EA significantly enhances cell viability following UVB exposure by mitigating apoptosis and preserving mitochondrial membrane potential (MMP). This protective effect is largely attributed to quercitrin and hyperoside, which, at 100 µM, reduce cleaved-PARP and cleaved-caspase-3 levels, suppressing apoptosis pathways. Additionally, HC-EA modulates inflammatory responses by downregulating IL-6, IL-8, COX-2, and iNOS expression while promoting Nrf2 activation and elevating HO-1 and SOD levels. Mechanistically, HC-EA inhibits the phosphorylation of p38 and JNK while enhancing ERK and Akt phosphorylation, thereby modulating the MAPK and Akt signaling pathways to enhance cellular resilience against UVB-induced oxidative stress [117].

In HDFs, HC-EA and its active components not only mitigate oxidative stress but also contribute to extracellular matrix (ECM) integrity by modulating collagen metabolism. At concentrations below 100 µg/mL, HC-EA significantly suppresses UVB-induced ROS production and reduces MMP-1 expression while simultaneously enhancing collagen synthesis. These effects are primarily mediated through the inhibition of ERK and JNK activation, leading to reduced AP-1 transcriptional activity and decreased MMP-1 expression. Furthermore, HC-EA suppresses the release of pro-inflammatory cytokines IL-6 and IL-8 at both the mRNA and protein levels [24].

Collectively, these findings underscore the potential of *H. cordata* extracts as natural photoprotective agents. By reducing oxidative stress, suppressing inflammatory responses, preventing apoptosis, and preserving ECM integrity, HC-EA and its active flavonoids offer promising therapeutic applications in the prevention of UV-induced skin aging and photodamage.

### 3.17. Malus baccata

*Malus baccata* (Siberian crab apple), a deciduous tree of the Rosaceae family, is rich in polyphenolic compounds, particularly protocatechuic acid and catechin, which exhibit strong antioxidant, antibacterial, and anticancer activities. Studies have demonstrated that *M. baccata* bark extract effectively scavenges free radicals and exerts cytotoxic effects against Jurkat leukemia cells. Additionally, it displays potent antibacterial activity against *Listeria monocytogenes*, *Bacillus cereus*, and *Escherichia coli*, as well as antifungal effects against *Aspergillus niger*, *Rhizopus nigricans*, and *Candida albicans*, with protocatechuic acid playing a key role in these protective mechanisms [118].

Recent studies have highlighted the protective effects of *M. baccata* methanol extract (Mb-ME) against UVB-induced oxidative stress and inflammation in human keratinocytes (HaCaT cells). In vitro, treatment with 100 µg/mL Mb-ME significantly reduces ROS generation and inhibits the expression of MMP-2, MMP-3, MMP-9, and IL-6, thereby preventing extracellular matrix (ECM) degradation and inflammatory responses. Moreover, Mb-ME enhances skin hydration by upregulating hyaluronan synthases (HASs) while suppressing hyaluronidases (HYALs), reducing hyaluronic acid breakdown. Increased expression of collagen type I alpha 1 chain (COL1A1) further contributes to ECM integrity, counteracting UVB-induced photoaging. Mechanistically, these effects are mediated through inhibition of the NF-κB and MAPK pathways, which are central regulators of inflammatory signaling and oxidative stress [119].

In addition to its protective role against oxidative stress, Mb-ME enhances the expression of essential skin barrier proteins, including transglutaminase 1 (TGM1) and filaggrin (FLG), strengthening the epidermal defense against environmental insults. Furthermore, in B16-F10 melanoma cells, Mb-ME suppresses melanogenesis by reducing melanin secretion and cellular melanin content, although it does not directly affect tyrosinase activity or the expression of key melanogenic regulators such as MITF and TYRP1. These findings suggest that Mb-ME modulates skin pigmentation through alternative pathways, making it a potential candidate for hyperpigmentation treatment [119].

Collectively, *M. baccata* exhibits significant photoprotective and skin-rejuvenating properties by mitigating oxidative stress, reducing inflammation, enhancing hydration, and preventing ECM degradation. Its ability to regulate key molecular pathways involved in skin aging and pigmentation underscores its potential as a multifunctional agent in dermatological applications.

### 3.18. Lonicera japonica

*Lonicera japonica* (honeysuckle), a perennial vine of the Caprifoliaceae family, has been widely used in traditional medicine for its antioxidant, anti-inflammatory, and skin-protective properties. The primary bioactive compound, chlorogenic acid, penetrates the skin effectively and mitigates UV-induced oxidative stress [120,121].

The antioxidant and cytoprotective effects of *L. japonica* have been extensively investigated in UV-exposed keratinocytes. Treatment with 0.5 mg/mL *L. japonica* aqueous extract significantly reduces ROS accumulation, DNA damage, and apoptosis in UVB-irradiated HaCaT cells. This protective effect is attributed to the high concentration of polyphenols and flavonoids, particularly chlorogenic acid, which enhances endogenous antioxidant defenses [122]. Additionally, a polyphenol-enriched composite extract containing *L. japonica* and Citrus reticulata (LCPE) enhances the suppression of UV-induced oxidative stress. By downregulating COX-2 and iNOS expression and modulating the MAPK/NF-κB signaling pathways, this composite extract provides superior cytoprotective effects in keratinocytes [123].

Beyond its antioxidant potential, *L. japonica* also demonstrates potent anti-inflammatory activity, contributing to its therapeutic benefits in UV-induced skin damage. In an LPS-induced inflammatory model, aqueous extract from *L. japonica* (AELJ) effectively prevents NF-κB p65 nuclear translocation and stabilizes IκBα levels, thereby suppressing the expression of pro-inflammatory mediators such as TNF-α and iNOS [124]. These findings suggest that *L. japonica* could alleviate UVB-induced cutaneous inflammation by modulating inflammatory signaling pathways.

Additionally, ethanol extracts of *L. japonica* exhibit strong radical-scavenging activity, inhibit tyrosinase function, and suppress melanin synthesis, contributing to its depigmenting effects [125]. *L. japonica* extract also downregulates MMP-1 expression while promoting collagen synthesis, thereby alleviating UV-induced wrinkle formation and preserving skin elasticity [126].

Collectively, *L. japonica* exhibits broad-spectrum protective effects against UV-induced skin damage by reducing oxidative stress, suppressing inflammation, and maintaining extracellular matrix integrity. Its bioactive constituents, particularly chlorogenic acid and isochlorogenic acids, hold significant potential for application in cosmeceuticals and dermatological formulations targeting photoaging and UV-related skin disorders.

## 4. Innovative Therapeutic Strategies for Skin Repair Using Medicinal Plants

Exosomes, hydrogels, and nanoemulsions represent cutting-edge strategies for enhancing the delivery and therapeutic potential of medicinal plant extracts in skin repair. These systems address critical challenges associated with conventional treatments, such as limited bioavailability, stability, and targeted delivery, by leveraging unique properties like nanoscale size, biocompatibility, and controlled release mechanisms. Exosomes excel in protecting and transporting bioactive molecules while facilitating cell communication and regeneration. Hydrogels provide sustained-release capabilities, high moisture retention, and improved stability for plant extracts, making them particularly effective in wound healing and infection prevention. Nanoemulsions, with their superior skin penetration and active ingredient protection, ensure deeper dermal delivery and enhanced photoprotective effects.

### 4.1. Exosomes

Exosomes are extracellular vesicles ranging in size from 30 to 150 nm, released into the extracellular environment via the fusion of multivesicular bodies (MVBs) with the plasma membrane. The lipid-rich outer membrane of exosomes encloses bioactive molecules, including proteins, RNA, microRNA, and DNA. Exosomes play pivotal roles in intercellular communication, immune response regulation, and tissue repair processes. Exosomes are abundant in human cells and are also produced by plant cells, where they influence diverse physiological processes by modulating signaling pathways [127,128,129].

**Figure 2:**
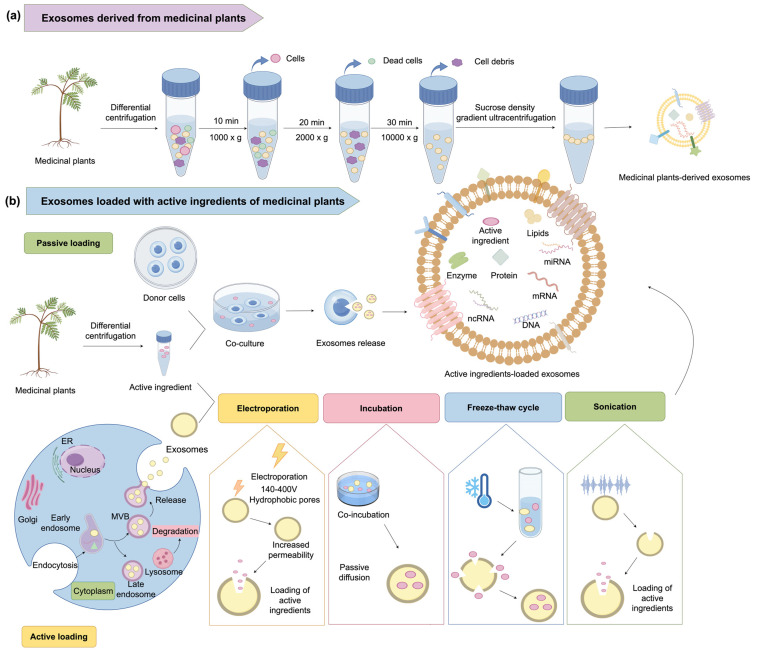
Isolation and loading strategies for medicinal plant exosomes. (**a**) Isolation of exosomes from medicinal plants. Exosomes are isolated from medicinal plants through differential centrifugation, followed by sucrose density gradient ultracentrifugation to ensure high purity. (**b**) Strategies for loading active ingredients into exosomes. Exosomes are loaded with active ingredients using passive or active loading methods. Passive loading involves co-culturing donor cells with active ingredients to facilitate their uptake, whereas active loading employs techniques such as electroporation, freeze–thaw cycles, or sonication to enhance the encapsulation efficiency of active ingredients.

In recent years, exosomes associated with medicinal plants have become a prominent research focus and can be classified into two types: exosomes directly isolated from medicinal plants and those loaded with plant active ingredients (Figure 2). The former preserves the biological activity of plant-derived active ingredients, enabling them to reduce inflammatory factor secretion, promote angiogenesis, stimulate collagen deposition, and regulate fibroblast proliferation and differentiation, effectively treating dermatitis, photoaging, and skin cancer [130,131]. The latter introduces active ingredients into exosomes through passive or active loading techniques. Passive loading involves co-culturing active ingredients with donor cells, leading to exosome secretion that naturally carries these active ingredients. Active loading, however, employs methods such as electroporation, co-incubation, freeze–thaw cycles, or sonication to directly incorporate active ingredients into exosomes [132]. This strategy significantly enhances the stability and bioavailability of active ingredients, paving the way for the modernization of medicinal plant applications.

Studies indicate that ginseng-derived exosomes, isolated via ultracentrifugation, can activate ERK and AKT/mTOR signaling pathways, promoting cell proliferation and expediting the repair of damaged skin [133]. Comparable results have been validated in exosomes derived from *S.baicalensis*, *A. membranaceus*, *P. suffruticosa*, and *C. asiatica*. Flavonoids encapsulated within *S. baicalensis*-derived exosomes demonstrate improved skin penetration while preserving their antioxidant and anti-inflammatory properties, making them effective in treating skin inflammation [134]. Asiaticide derived from *C. asiatica*, when incorporated into exosomes, enables its active ingredients to penetrate deeper skin layers and enhance skin structure and elasticity by stimulating collagen synthesis [135]. Additionally, *C. asiatica* exosomes exhibit remarkable efficacy in wrinkle reduction, skin regeneration, and anti-inflammatory activity [133]. Extracts of *A. membranaceus* loaded into mesenchymal stem cell-derived exosomes effectively suppress the expression of inflammatory factors, promote wound healing, and minimize scar formation by curbing abnormal fibroblast proliferation [136]. Paeonol-loaded transethosomes derived from *P. suffruticosa*, prepared via ethanol injection, effectively enhance the solubility and stability of paeonol while mitigating its volatility. Compared to conventional vesicles, transethosomes exhibit superior encapsulation efficiency, promote higher transdermal flux and skin deposition, and prolong systemic circulation, thereby significantly improving the bioavailability and therapeutic potential of paeonol for treating inflammatory skin conditions [137].

### 4.2. Hydrogels

Hydrogels, as three-dimensional hydrophilic polymer networks, are widely applied in wound care due to their ability to maintain wound moisture, prevent infections, and exhibit excellent biocompatibility. By integrating medicinal plant extracts, hydrogels serve as a sustained-release drug delivery system, significantly enhancing drug targeting and therapeutic efficacy. The structure of hydrogels is formed through either physical cross-linking (e.g., hydrogen bonding, van der Waals forces) or chemical cross-linking (e.g., covalent bonds), enabling them to absorb large amounts of water and create a gel-like consistency resembling biological tissues. This compatibility allows hydrogels to act as carriers for plant extracts, including flavonoids, polyphenols, and alkaloids, which are either embedded within the gel matrix or encapsulated in nanocarriers such as liposomes or nanocapsules before integration [138,139,140,141].

The release of active ingredients from hydrogels is primarily diffusion-driven and can be modulated by altering the hydrogel’s cross-linking density, pore size, and network composition. Hydrogels also offer tunable responsiveness to environmental stimuli, such as pH, temperature, and light, enabling controlled and intelligent drug delivery. Advantages of hydrogels include their high biocompatibility, ability to maintain skin hydration, controlled release of active ingredients, enhanced skin penetration, and protection of plant extracts from environmental degradation [138,139,140,141].

Hydrogels have demonstrated great promise in the treatment of skin damage by leveraging these advantages. For instance, integrating *Paeonia suffruticosa* extract with the thermosensitive polymer Poloxamer 407 improved drug diffusion, effectively mitigating skin inflammation [142]. Similarly, *Plantago* extract combined with carboxymethyl chitosan (CMCS) hydrogel exhibited potent anti-inflammatory and antioxidant effects, significantly promoting collagen deposition and preventing wound infections [143]. Hydrogels prepared using *Centella asiatica* extract showed robust antibacterial properties, and unlike antibiotics, they did not provoke resistance, making them suitable for prolonged use [144].

Several advanced applications highlight the potential of hydrogel-based systems in treating UV-induced skin damage. Dihydromyricetin (DMY), a flavonoid encapsulated in cationic nanocapsules and incorporated into hydrogels, exhibited significant photoprotective and antioxidant effects. DMY demonstrated 50% SPF UVB protection, reduced DNA lesions by 99.9%, and effectively scavenged ROS induced by solar radiation [145]. Silibinin, derived from milk thistle, showed strong anti-inflammatory effects when delivered through nanocapsule-enriched hydrogels, reducing UVB-induced skin damage in mice [146]. Hydrogels containing asiatic acid (AA) from *C. asiatica*, combined with enhancers such as L-menthol and diethylamine, facilitated superior skin penetration and release rates, significantly improving skin barrier repair [147]. Furthermore, liposome-enriched hydrogels containing Tetramethylpyrazine (TMPZ) from *Ligusticum wallichii* demonstrated in vivo efficacy in reducing skin thickness, wrinkles, and erythema caused by UV exposure [148]. Additional plant-based hydrogel applications include formulations incorporating sulfated galactofucan polysaccharides (SGPs) and alginate oligosaccharides (AOSs) from marine algae, which exhibit antioxidant, anti-inflammatory, and collagen-preserving properties for mitigating UVB-induced photoaging [149]. Rosemary (*Rosmarinus officinalis*) extract-loaded emulgel has also been demonstrated to prevent UVB-induced skin damage by reducing oxidative stress and inflammation [150].

### 4.3. Nanoemulsions

Nanoemulsions, comprising oil, water, surfactants, and co-surfactants, are colloidal dispersion systems with particle sizes ranging from 10 to 1000 nm. These systems leverage their nanometric scale to significantly enhance the permeability of active ingredients through the skin, facilitating targeted delivery to deeper layers. Nanoemulsions are widely recognized for their superior stability, ability to protect active ingredients from environmental degradation, and potential to improve the bioavailability of both hydrophilic and lipophilic compounds. By forming a thin, occlusive film on the skin, nanoemulsions maintain hydration and ensure even distribution of active ingredients. Additionally, their low viscosity makes them user-friendly and easy to apply [151,152,153].

In the context of skin repair, nanoemulsions exhibit notable advantages in delivering medicinal plant extracts due to their controlled release and enhanced penetration capabilities. For instance, asiaticoside and paeoniflorin, when loaded into nanoemulsions, demonstrate improved skin barrier repair and potent anti-inflammatory effects [154]. Several studies further highlight the efficacy of nanoemulsions in treating UV-induced skin damage. Nanoemulsions encapsulating resveratrol effectively penetrate deeper skin layers to counteract UV-induced oxidative stress [155]. Another study on catechin-loaded nanoemulsion gels reported promising photoprotective properties against UVA-induced oxidative damage in keratinocytes [156]. Additionally, nanoemulsions developed using the ethyl acetate fraction (EAF) of pomegranate seed oil (PSO) exhibited photoprotective effects against UVB-induced DNA damage in HaCaT keratinocyte cell lines [157]. Recent studies have also explored nanoemulsions incorporating Buriti oil (*Mauritia flexuosa*) and *Aloe vera* extract, which exhibit excellent photoprotective and moisturizing properties. This formulation (NE-A19) demonstrated broad-spectrum UVA/UVB protection. Moreover, NE-A19 enhances skin hydration and occlusion, surpassing nanoemulsions lacking *Aloe vera* [158].

Collectively, these advanced delivery systems bridge traditional phytomedicine and modern drug delivery technologies. They not only amplify the efficacy of medicinal plant extracts in treating UV-induced skin damage but also pave the way for innovative therapeutic solutions in dermatology, providing promising directions for future interdisciplinary research and clinical applications.

## 5. Challenges and Prospects of Medicinal Plants and Their Delivery Technologies

With the advancement of biological sciences and medicine, medicinal plants have demonstrated significant potential in treating skin diseases owing to their low toxicity, abundant availability, and unique therapeutic properties. However, to fully realize their clinical value and facilitate modernization, several challenges must be addressed. Below are the key research priorities and future directions in this domain.

### 5.1. Component Analysis and Identification of Medicinal Plant Extracts

Although the therapeutic benefits of medicinal plants are widely recognized, the analysis and identification of their specific active ingredients remain a fundamental challenge. The bioactive compounds in many plant extracts are not yet fully characterized, hindering both mechanistic studies and the standardization of medicinal plant production. Furthermore, current research predominantly focuses on isolating single compounds, which often overlooks the synergistic effects of multi-component interactions within medicinal plants. Consequently, advancing multi-component extraction techniques and integrated pharmacological methodologies will be a pivotal direction for future breakthroughs [159].

### 5.2. Establishment of Quality Control Standards for Medicinal Plants

The chemical composition and therapeutic efficacy of medicinal plants are profoundly influenced by factors such as species, origin, ecological environment, growth stage, medicinal part, processing methods, and storage conditions. These variables result in challenges to maintaining the stability and consistency of drug quality, ultimately impacting clinical effectiveness. To address these issues, developing robust quality control standards and employing modern analytical technologies—such as high-performance liquid chromatography (HPLC) and mass spectrometry—will enable precise qualitative and quantitative assessments of key constituents, thereby enhancing the clinical reliability of medicinal plants.

### 5.3. In-Depth Research on Medicinal Plant-Derived Exosomes

Medicinal plant-derived exosomes are gaining attention as a novel delivery system, demonstrating immense potential due to their stable origins, minimal side effects, and low immunogenicity. However, current research is predominantly at the exploratory stage, with the molecular mechanisms of exosome-mediated signal pathway modulation and pharmacological effects yet to be fully elucidated. Additionally, methods for their isolation, characterization, and efficient loading of active ingredients remain underdeveloped. Refining exosome loading strategies, including passive and active techniques, will be a key focus for future investigations [132].

### 5.4. Synergistic Use of Stem Cell-Derived Exosomes and Medicinal Plants

The active ingredients of medicinal plants can be incorporated into mesenchymal stem cell (MSC)-derived exosomes, merging the anti-inflammatory and antimicrobial effects of medicinal plants with the tissue repair properties of stem cell exosomes to greatly enhance therapeutic efficacy. This innovative approach not only accelerates the healing of skin injuries but also minimizes scar formation. However, sourcing MSC-derived exosomes from bone marrow, adipose tissue, or umbilical cord blood is limited and cost-intensive. While stem cell exosomes exhibit excellent biocompatibility and low immunogenicity, immune rejection may still occur during allogeneic transplantation. Enhancing the methodologies for stem cell exosome extraction and application remains a pivotal direction for future research [136].

### 5.5. Bridging the Gap Between Basic Research and Clinical Applications

The development of new therapeutics often spans up to two decades, encompassing phases from drug design and active ingredient identification to animal studies, clinical trials, and eventual regulatory approval. At present, most studies on medicinal plant-derived exosomes remain at the preclinical stage, with only a few advancing to early-phase clinical trials. As reported by ClinicalTrials.gov, by May 2024, a total of 192 exosome-related clinical studies had been registered globally, primarily focusing on cancer and COVID-19, with only 10 targeting skin conditions, most of which are still in Phase I trials. Accelerating the clinical application of medicinal plant-derived exosomes requires innovations in exosome isolation and characterization techniques, broader research on medicinal plants, and in-depth elucidation of the molecular targets and mechanisms of their active ingredients [127].

### 5.6. Integration of Advanced Drug Delivery Technologies

Needle-free injection, a novel drug delivery system, has gained significant attention in recent years. This technology is already utilized in diabetes management, particularly for insulin delivery without needles. Looking ahead, combining needle-free injection with formulations such as medicinal plant-derived exosomes could leverage high-pressure jet streams to enable rapid skin penetration and deep tissue delivery. This integration promises to greatly enhance drug permeability and targeting, providing a more effective approach for treating skin injuries [160].

## 6. Conclusions

In recent decades, the extensive use of chlorofluorocarbon compounds and escalating environmental pollution have accelerated ozone layer depletion, exposing the Earth’s surface to increased ultraviolet (UV) radiation and posing significant threats to human skin health. Prolonged UV exposure causes sunburn and photoaging and increases the risk of severe conditions, including skin cancer. Medicinal plants, recognized for their safety and diverse therapeutic potential, have become a promising strategy to mitigate and manage UV-induced skin damage.

This review provides a comprehensive analysis of the origins, active ingredients, and therapeutic mechanisms of 18 medicinal plants in combating UV-induced skin damage. Evidence suggests that these plants mitigate UV-related harm through various molecular pathways, including the suppression of inflammatory responses, attenuation of oxidative stress, enhancement of DNA repair, and regulation of melanin deposition. Additionally, modern drug delivery systems, such as exosomes, hydrogels, and nanoemulsions, have substantially increased the stability and bioavailability of active ingredients, boosting their penetration and targeting in deeper skin layers, thereby enhancing therapeutic outcomes.

While the potential of medicinal plants in managing UV-induced skin damage is widely recognized, numerous aspects warrant further investigation. Key priorities include the accurate identification of active ingredients, understanding multi-component synergistic mechanisms, and refining standardized production techniques for plant-derived materials. Furthermore, the extraction and loading technologies for medicinal plant exosomes are still in their nascent stages, and detailed studies on their molecular functions and clinical applications remain imperative.

In summary, the integration of medicinal plants with advanced drug delivery systems represents a cutting-edge approach to combating UV-induced skin damage. Future multidisciplinary studies focusing on molecular targets and therapeutic mechanisms, while refining delivery technologies, are expected to expedite the development of medicinal plants into innovative therapeutic agents, opening up new horizons for managing skin-related diseases.

## Figures and Tables

**Table 1:** Key active ingredients and mechanisms of medicinal plants in repairing UV-induced skin damage.

Medicinal Plants	Active Ingredients	In Vitro Models	Concentration in Cell Culture Media	In Vivo Models	Dose (Route of Administration)	Mechanisms	Signaling Pathways	Ref.
*Salvia* *miltiorrhiza* (Danshen)	Danshensu	α-MSH- stimulated B16 melanoma cells	2 mM	N.A.	N.A.	↓: IL-6, IL-8, MMP-1, MMP-3	Nrf2 AMPK/SIRT1/PGC-1α	[41,42,43,44,45,46]
	Salvianolic acid B	α-MSH- stimulated B16 melanoma cells	0.5 mM	N.A.	N.A.			
	Cryptotanshinone	HaCaT cells exposed to UVB (200 mJ/cm^2^)	0.1 μM	N.A.	N.A.			
		HFF-1 cells exposed to UVA (15 J/cm^2^).	0.1 μM	N.A.	N.A.			
*Panax* *notoginseng* (Sanqi)	Protopanaxtriol saponins(Rg1, Re, R1)	MSH-stimulated B16 cells	0.1 mg/mL	UVB-irradiated C57BL/6 mice	10 mg/mL (Topical application)	↑: LL-37 ↓: PGE-2, TNF-α, IL-1β	P13K/AKT	[47,48,49,50,51]
	Panax notoginseng saponins	HaCaT cells exposed to UVB (100 mJ/cm^2^)	200 μg/mL	N.A.	N.A.			
*Astragalus* *membranaceus* (Huangqi)	Polysaccharides	HaCaT cells exposed to UVA (30 mJ/cm^2^)	500 μg/mL	N.A.	N.A.	↑: SOD ↓: TLR4, IL-6, MDA, ROS, MMP-1	TLR4/NF-κB	[52,53,54,55,56,57]
	Astragaloside IV	HaCaT cells exposed to UVB (50 mJ/cm^2^)	50 μM	N.A.	N.A.			
*Andrographis paniculata* (Green chiretta)	Andrographolide	N.A.	N.A.	UVB-irradiated mice (180 mJ/cm^2^)	3.6 mg kg/b.wt (Topical application)	↑: Collagen ↓: GSH, ROS, TBARS, CXCL1, IL-6, IL-1β, TNF-α	PI3K/Akt	[58,59,60,61,62,63,64]
*Paeonia* *lactiflora* (Chinese peony)	Paeoniflorin	α-MSH-stimulated B16-F10 cells	10 μM	N.A.	N.A.	↓: COX-2, IL-6, TNF-α, DPPH, MITF, TYRP-1, TYRP-2	MAPK/ERK ROS/P38/P53	[65,66,67,68,69,70,71,72]
	Paeonol	JB6 Cl41 cells exposed to SUV (20 KJ/m^2^)	400 μM	N.A.	N.A.			
		HaCaT cells exposed to SUV (20 KJ/m^2^)	400 μM	N.A.	N.A.			
*Panax* *ginseng* (Ginseng)	Ginseng oligosaccharide	HaCaT cells exposed to UVB (40 mJ/cm^2^)	20 μg/mL	UVB-irradiated BALB/c hairless mice (200 mJ/cm^2^)	1.0 mg/cm^2^/day	↑: FLG, IVL, AQP3, VMP1, SPINK5, DSG1 ↓: KLK5, KLK7, MMP-1, MMP-2, MMP-3, MMP-13, TGM1, COX-2	MAPK/ERK NF-κB PI3K/AKT	[73,74,75,76,77,78]
	Panax ginseng extract	HaCaT cells exposed to UVB (6 mJ/cm^2^)	40 μg/mL	UVB-irradiated BALB/c hairless mice (300 mJ/cm^2^)	4 mg/g (Oral administration)			
	Panax ginseng C. A. Meyer extract	N.A.	N.A.	UVB-irradiated BALB/c hairless mice (200 mJ/cm^2^)	2 mg/cm^2^ (Topical application)			
*Scutellaria* *baicalensis* (Chinese skullcap)	Baicalin	HaCaT cells exposed to UVB (5 mJ/cm^2^)	100 μM	UVB-irradiated hairless mice (200 mJ/cm^2^)	50 mg kg/b.wt (Oral administration)	↓: MMP-9, VEGF, NF-κB/p65, HIF-1α, COX-2	Nrf2 NF-κB RDMAPK/ERK	[79,80,81,82]
	Wogonin	HaCaT cells exposed to UVB (5 mJ/cm^2^)	100 μM	UVB-irradiated hairless mice (200 mJ/cm^2^)	50 mg kg/b.wt (Oral administration)			
*Rhodiola rosea* (Golden root)	Salidroside	HaCaT cells exposed to UVB (25 mJ/cm^2^)	100 μM	N.A.	N.A.	↑: Beclin-1, ATG7 ↓: P62, MMP-2, IL-6, IL-10, IL-1β, TNF-α	NF-κB Nrf2/Keap1	[83,84,85,86,87]
	Rosavin	HaCaT cells exposed to UVB (7.6 mW/cm^2^) and UVA (21.4 mW/cm^2^)	50 μg/mL	N.A.	N.A.			
*Tripterygium wilfordii* (Thunder god vine)	Celastrol	B16-F10 melanoma cells	10 μM	N.A.	N.A.	↓: IL-17A, IL-23, HIF-1α, STAT3, TNF-α	NF-κB PI3K/AKT/mTOR	[88,89,90,91,92]
*Paeonia* *suffruticosa* (Moutan peony)	Paeonol	HaCaT cells exposed to UVB (125 mJ/cm^2^)	10 μg/mL	UVB-irradiated albino hairless mice (125 mJ/cm^2^)	0.1% paeonol	↑: NQO-1, DLD, Collagen, HO-1, NQO-1 ↓: MMP-1	MAPK/AP-1 Nrf2	[93,94]
*Centella* *asiatica* (Gotu kola)	Asiatic acid glucosamine salt(AAGS)	Human dermal fibroblasts exposed to UVB (200 mJ/cm^2^)	25 μg/mL	UVB-irradiated nude mice (270 mJ/cm^2^)	60 mg/mL (Topical application)	↑: Collagen ↓: MMPs, ROS	MAPK/AP-1 Ras	[95,96,97,98,99]
	Titrated extract of Centella asiatica (TECA)	HaCaT cells exposed to UVB (50 mJ/cm^2^)	5 μg/mL	N.A.	N.A.			
*Arctium lappa* (Greater burdock)	Arctiin	NHDF cells exposed to UVB (100 mJ/cm^2^)	10 μM	N.A.	N.A.	↑: miR-205-3p, SIRT6 ↓: miR-3652, miR-513a-5p, miR-1290, miR-378b	MAPK/ERK NF-κB	[100,101,102,103]
*Aloe vera* (Aloe)	Fermented Aloe vera extract	α-MSH-stimulated B16-F10 cells	10 μM	10 μM	10 μM	↓: MITF, TYRP-1, TYRP-2 TYR↓	MAPK/Rho TGF-β	[104,105,106,107]
*Sophora* *flavescens* (Shrubby sophora)	Kushenol C	N.A.	N.A.	UVB-irradiated SHK-1 mice (120 mJ/cm^2^)	10 mg kg/b.wt (Oral administration)	↑: GSH, SOD, CAT ↓: IL-8, TNF-α, MDA, c-KIT	MAPK/ERK PI3K/AKT NF-κB	[108,109,110,111,112]
	Kuraridin	α-MSH-stimulated B16-F10 cells	5 μM	N.A.	N.A.			
*Saussurea* (Snow lotus)	The ethanol extract of *Saussurea laniceps*	N.A.	N.A.	Mice exposed to UVB (20.52 J/cm^2^) and UVA (142.50 J/cm^2^)	360 mg kg/b.wt (Oral administration)	↑: SOD, GSH-PX, HYP ↓: γ-H2AX, CPDs, TNF-α, IL-1β	PI3K/Akt PPAR-α	[113,114,115]
	Saussurea involucrata polysaccharide	HaCaT cells exposed to UVB (200 mJ/cm^2^)	50 μg/mL	C57BL/6 mice were exposed to UVB (500 mJ/cm^2^)	0.5% (Topical application of drug gel)			
*Houttuynia* *cordata* (Fish mint)	Quercitrin	HaCaT cells exposed to UVB (15 mJ/cm^2^)	100 μM	N.A.	N.A.	↑: HO-1, SOD ↓: TNF-α, IL-6, IL-1β, COX-2, iNOS, IL-8, MMP-1	MAPK/ERK PI3K/AKT NF-κB Nrf2	[116,117]
	Hyperoside	HaCaT cells exposed to UVB (15 mJ/cm^2^)	100 μM	N.A.	N.A.			
		Human dermal fibroblasts exposed to UVB (15 mJ/cm^2^)	20 μg/mL	N.A.	N.A.			
*Malus baccata* (Siberian crab apple)	The methanol extract of *Malus baccata*	α-MSH-stimulated B16-F10 cells	100 μg/mL	N.A.	N.A.	↑: FLG, TGM1, HAS-1, HAS-2, HAS-3, Collagen ↓: TNF-α, IL-6, IL-1β, COX-2, HYAL-1, HYAL-4	MAPK/ERK NF-κB PI3K/AKT	[118,119]
		HaCaT cells exposed to UVB (30 mJ/cm^2^)	100 μg/mL	N.A.	N.A.			
		Human dermal fibroblasts exposed to UVB (30 mJ/cm^2^)	100 μg/mL	N.A.	N.A.			
*Lonicera* *japonica* (Honeysuckle)	Chlorogenic Acid	HaCaT cells exposed to UVB (30 mJ/cm^2^)	0.5 mg/mL	N.A.	N.A.	↑: Collagen ↓: MMP-1, DPPH, ROS, COX-2, iNOS	MAPK/ERK NF-κB	[120,121,122,123,124,125,126]

N.A. indicates the absence of reported in vitro or in vivo studies. Arrows (↑, ↓) denote the increase or decrease of gene expression, protein levels or biochemical markers induced by plant-derived compounds.
